# Electroacupuncture Improves Bladder and Bowel Function in Patients with Traumatic Spinal Cord Injury: Results from a Prospective Observational Study

**DOI:** 10.1155/2013/543174

**Published:** 2013-12-07

**Authors:** Zhishun Liu, Weiming Wang, Jiani Wu, Kehua Zhou, Baoyan Liu

**Affiliations:** ^1^Department of Acupuncture, Guang An Men Hospital, China Academy of Chinese Medical Sciences, No. 5 Bei Xian Ge Street, Xicheng, Beijing 100053, China; ^2^Beijing University of Chinese Medicine, No. 11 North Third Ring Road East, Chaoyang, Beijing 100029, China; ^3^Department of Health Care Studies, Daemen College, 4380 Main Street, Amherst, NY 14226, USA

## Abstract

In order to explore the effect of electroacupuncture (EA) for chronic bowel and bladder dysfunction after traumatic spinal cord injury, 14 patients were treated with electroacupuncture once a day, five times a week for the first four weeks, and once every other day, three times a week for the following four weeks. The patients were then followed up for six months. After treatment, four (4/14, 28.57%) patients resumed normal voiding; six (6/14, 42.86%) resumed normal voiding for no less than half of all micturition behaviors; four (4/14, 28.57%) required supplementary urination methods for higher than half of all micturition behaviors. These effects persisted during followup. Mean postvoid RUV decreased by 190.29 ± 101.87 mL (*P* < 0.01) after treatment and by 198.86 ± 112.18 mL (*P* < 0.01) during followup. Patients' weekly urinary incontinence frequency decreased 7.14 ± 46.34 times/week (*P* = 0.036) after treatment and decreased 49.86 ± 44.38 times/week during followup. After treatment, four (4/14, 28.57%) patients resumed normal bowel movements (*P* = 0.025); five (5/14, 35.71%) reduced the dependence on supplementary defecation methods; five (5/14, 35.71%) had no changes. In patients with chronic bowel and bladder dysfunction after traumatic SCI, EA may provide a valuable alternative tool in improving patients' self-controlled bowel and bladder functions with minimal side effects.

## 1. Introduction

Traumatic spinal cord injury (SCI) is an accidental disaster, causing unexpected suffering physically, emotionally, and costly to patients [1]. Traumatic SCI can cause disorders of somatesthesia and locomotion below the level of injury. Neurogenic bladder and bowel dysfunctions which affect the physical and psychological health of patients and severely decrease the quality of life are also prevalent in patients with traumatic SCI. Urological complications of neurogenic bladder caused by traumatic SCI consist of inability to empty the bladder, urinary tract infections (UTIs), incontinence, and upper urinary tract deterioration [2]. A distended bladder may also cause pressure effects on adjacent structures including the left common iliac vein in the pelvis leading to adverse events of other organs [3]. Common bowel dysfunctions in patients with SCI may include poorly localized abdominal pain, difficulty with bowel evacuation, hemorrhoids, abdominal distention, and autonomic hyperreflexia arising from the gastrointestinal tract [4]. In patients with SCI, treatments for bladder dysfunctions may involve conservative treatments (drug treatments, catheterization, assisted bladder emptying, rehabilitation, and external appliances), minimally invasive treatments (botulinum toxin A injections in bladder, intravesical vanilloid treatment, sphincterotomy, and ureteral reimplantation), and surgical treatments (autoaugmentation, sacral anterior root stimulation, sacral neuromodulation, and artificial urinary sphincter) [5]; treatments for bowel dysfunctions may include nonpharmacological therapies (suppositories, reflex stimulation, abdominal massage, assistive devices, and others), pharmacological agents (prucalopride, metoclopramide, neostigmine, and others), and surgical interventions (implantation of electrical stimulation systems, colostomy, antegrade continence enema, and enema continence catheter) [6]. Although these treatments are effective to some extent, none can help patients regain the self-controlled bladder and bowel function. Furthermore, treatments like catheterization, sacral anterior root stimulation, colostomy, and enema will likely cause additional pain and other side effects to patients. Therefore, an effective and safe treatment with few side-effects is warranted for the treatment of bladder and bowel dysfunction in patients with traumatic SCI.

Acupuncture is a therapy that is commonly utilized by the Chinese people for the management of bowel and bladder dysfunction. Documentation of acupuncture use for bowel and bladder symptoms dates back to thousands of years ago in the ancient medical monograph Huang Di Nei Jin (Yellow Emperor's Inner Canon). Propositions of therapeutic effects of acupuncture in neurogenic bowel and bladder dysfunctions are supported by modern research studies. Compared with urinary catheterization or use of medications, Cheng et al. [7] found that electroacupuncture (EA) could help acute SCI patients with neurogenic bladder achieve balanced (continent, catheter-free) bladder function, and Wong et al. [8] demonstrated that EA at SI3 and BL62 in conjugation with auricular acupoints promoted bladder function recovery in patients with acute SCI. In addition, Wong et al. [8] also found that EA could decrease the need with bowel care and episodes of fecal incontinence due to SCI. Similarly, manual acupuncture was found to induce resolution of urinary incontinence in 15% of patients with SCI-induced detrusor hyperreflexia [9]. In addition, the results of our previous case series study [10] and the study by Honjo et al. [9] indicate that EA could improve voiding abilities in patients with SCI. Among the aforementioned research studies, effects of EA on both bowel and bladder functions were only reported in the study by Wong et al. [8]. Nonetheless, due to that the main purpose was patients' general functional recovery after SCI, Wong et al. [8] did not provide sufficient information (such baseline values of patients' voiding abilities) regarding patients' bowel and bladder function. As quality of these acupuncture studies is of dispute, conclusive evidence regarding acupuncture effects in patients with simultaneous neurogenic bowel and bladder dysfunction after traumatic SCI is still lacking [11]. Consequently, in the present study, we aimed to explore the effects of EA for chronic bowel and bladder dysfunction after traumatic SCI.

## 2. Materials and Methods

### 2.1. Study Design

This was a prospective consecutive case series study performed at the Acupuncture Department of Guang An Men Hospital, China Academy of Chinese Medical Sciences. The hospital ethics committee approved this treatment protocol, and all patients signed informed consent before study participation. Acupuncture procedures were implemented by senior acupuncturists at the department with more than 20 years' clinical experiences. Data management and analysis were performed by graduates who were blinded to the treatment procedures.

### 2.2. Participants

For inclusion, the participants had to fulfill the following criteria: (1) simultaneous bladder and bowel dysfunctions caused by traumatic SCI of or above Level L1; (2) no history of sphincter injury or surgery of the urinary tract, gastrointestinal tract, or anus; (3) SCI history for no less than six months and onset of bowel and bladder symptoms after SCI; (4) they must be at least 18 years old; (5) cardiac, hepatic, and renal functions as well as coagulative functions are normal or near normal; (6) no cognitive or mental disorders.

### 2.3. Acupuncture Protocol

Huatuo brand needles (size 0.45 mm × 125 mm and 0.30 mm × 75 mm, manufactured by Suzhou Medical Appliance, Suzhou, Jiangsu, China) together with GB6805-2 Electro-Acu Stimulators (Medical Supply & Equipment Co., Ltd., Shanghai, China) were used. The parameters of electric stimulation were set as the follows: continuous wave with electric current frequency of 20 Hz and intensity between 3 and 10 mA according to patients' tolerance. Based on the clinical experiences and anatomical knowledge (direct stimulation of S2-S3) of the acupuncturists, bilateral BL32, BL33, and BL35 were used.

Acupoints were selected and localized according to the WHO Standardized Acupuncture Points Location [12]. Needles of the size of 0.30 mm × 75 mm were inserted into bilateral BL35 vertically with a depth of 45–75 mm; 0.45 mm × 125 mm size needles were inserted inwardly and downwardly into bilateral BL32 and BL33 (S2 and S3 foramina) at an angle of 20–30° with a depth of 80–90 mm. Paired alligator clips with negative and positive electrodes of the EA apparatus were attached to the needle holders at each pair of the same acupoints on each side during treatment. The positive electrodes were attached on the left, and the negative electrodes were attached on the right. Each EA treatment lasted for 50 minutes. All patients received EA treatment once a day, five times a week for the first four weeks, and once every other day, three times a week for the following four weeks. The patients were then followed up for 6 months.

### 2.4. Outcome Measurement

Bladder functions including postvoid residual urine volume (RUV), weekly urinary incontinence frequency, and additional conservative interventions (if any) were measured and documented at baseline, after treatment, and during followup. With reference to the International Lower Urinary Tract Function Basic Spinal Cord Injury Data Set [13], normal voiding in the present study is defined as voluntary initiation of micturition without reflex stimulation or compression of the bladder. Based on patients' voiding abilities during the past 24 hours, they were divided into three groups after treatment: patients who had normal voiding all the time were categorized into Group A; patients with normal voiding frequency no less than half of all micturition behaviors were categorized into Group B; all the remaining patients who require assistive urination help with a frequency for higher than half of all micturition behaviors were categorized into Group C.

With reference to the International Bowel Function Basic Spinal Cord Injury Data Set [14], patients' bowel movements were divided into normal, partially normal, and complete constipation. In the present study, normal bowel movement is defined as self-initiated bowel movements without any help of supplementary methods during the past 48 hours; partially normal bowel movement is defined as more than half of the times that patient have self-initiated bowel movements with occasional supplementary defecation method use during the past 48 hours; complete constipation is defined as more than half of the times that patient require supplementary defecation method use for bowel movements during the past 48 hours. Postvoid RUV was measured using a cylinder via self-urethral catheterization after urination. The frequencies of normal voiding and urinary incontinence, postvoid RUV, and bowel movement conditions, as well a supplementary urination and defecation methods were recorded by our research staff in a bladder and bowel diary each time during patients' visit.

### 2.5. Safety Measurement

Adverse events which may include hematoma, fainting, and unbearable pain and others were documented during study.

### 2.6. Statistical Analysis

Statistical analysis was performed with the SPSS software package (Version 17.0) for Windows XP. Quantitative data of postvoid RUV and weekly urinary incontinence frequency were expressed with mean ± standard deviations (SD). Paired samples *t*-test was used to measure the difference among values at baseline, after treatment, and during followup. A significance level of *P* < 0.05 and two-tailed tests were used for all analyses.

## 3. Results

**Figure 1:**
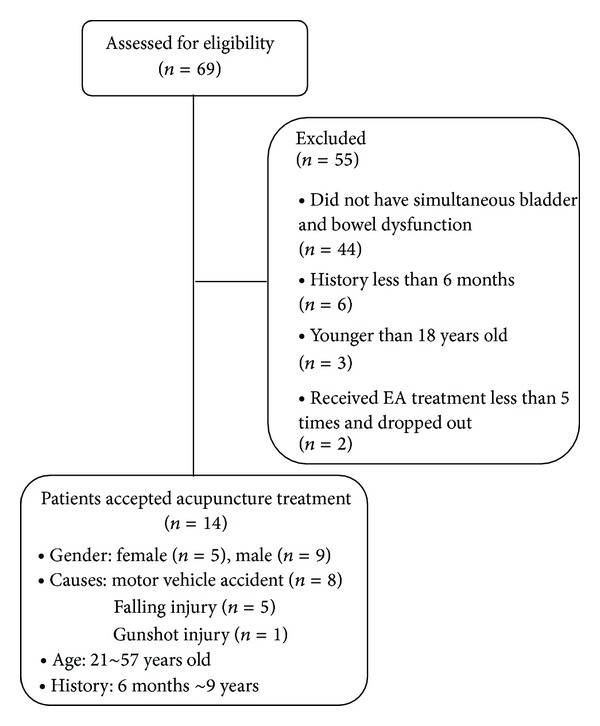
Flow chart of study participation.

As presented in Figure 1, from December 12, 2007, to October 31, 2011, a total of 69 patients with SCI visited the Outpatient Department of Acupuncture at Guang An Men Hospital. Among these patients, 25 had bladder and bowel dysfunctions; however, 11 of them were excluded from the present study for the following reasons: six patients had SCI injury less than six months ago; three patients were younger than 18 years old; two patients received EA treatment less than 5 times and dropped out during treatment. Consequently, 14 patients were included in the final analysis.

Of these 14 patients, nine were male and five were female; eight had motor vehicle accident, five were injured from falling, and one was injured by gun shot. The mean age of these participants was 34 years old with a range from 21 to 57 years old. The mean disease course was 16.3 months with a range from six months to nine years. In these 14 patients, two patients' injuries were at the cervical level, two at the upper thoracic level, four at the lower thoracic level, and six at the lumbar level. According to American Spinal Cord Injury Association (ASIA) Impairment Scale (AIS) classification [15], impairments of the included patients were classified as follows: five had Grade A (complete) impairment, six had Grade B (sensory incomplete) impairment, and three had Grade C (motor incomplete) impairment (Table 1).

### 3.1. Assessments of Bladder Function

Before treatment, all 14 patients had no normal voiding ability and required assisted therapies (intermittent catheterization or abdominal push) to empty the bladder. After eight-week treatment, four (4/14, 28.57%) patients resumed normal voiding (Group A); six (6/14, 42.86%) resumed normal voiding for no less than half of all micturition behaviors (Group B); four (4/14, 28.57%) required assistive urination therapies for higher than half of all micturition behaviors (Group C). In the six patients of Group B, three patients required bladder emptying via abdominal push, two required intermittent catheterization, and one required both. Similar results were found during followup (Table 1).

**Figure 2:**
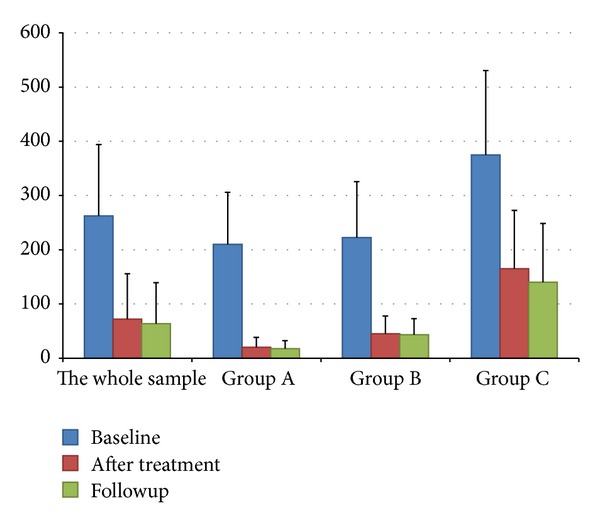
Histogram of mean postvoid RUV. Group A refers to patients who had normal voiding all the time; Group B refers to patients with normal voiding frequency no less than half of all micturition; Group C refers to patients who require assistive urination help with a frequency higher than half of all micturition.

Mean postvoid RUV for these 14 patients decreased by 190.29 ± 101.87 mL after treatment, from 262.43 ± 131.50 mL at baseline to 72.14 ± 83.59 mL (*P* = 0.000). Compared with baseline, the mean postvoid RUV was decreased by 198.86 ± 112.18 mL during followup, from 262.43 ± 131.50 mL at baseline to 63.57 ± 75.71 mL (*P* = 0.000). Compared with baseline, the mean postvoid RUV of patients was decreased by 190 ± 108.62 mL after eight-week treatment and by 192 ± 107.82 mL during followup in Group A; the decreases were 177.33 ± 112.01 mL after treatment and 179.00 ± 110.70 mL during followup in Group B, and 202.50 ± 118.43 mL after treatment and 220.00 ± 163.10 mL during followup in Group C (see Figure 2 and Table 2).

Of these 14 patients, seven patients (7/14, 50.00%) had various degrees of urinary incontinence. Five out of these seven patients were diagnosed as detrusor hyperreflexia through urodynamic study (UDS) before treatment; the remaining two patients lacked relevant examination. After treatment, the weekly urinary incontinence frequency for these seven patients reduced from 79.14 ± 64.80 times/week at baseline to 32.00 ± 20.94 times/week (*P* = 0.036); during followup, the weekly urinary incontinence frequency reduced to 29.28 ± 22.71 times/week (*P* = 0.025). Among the remaining seven patients who had urinary retention before treatment, four patients were diagnosed as detrusor areflexia through UDS, and the other three patients did not provide UDS information. During treatment, five (all four patients with detrusor areflexia and one with insufficient UDS information) out of these seven patients with urinary retention developed urinary incontinence. Two patients developed urinary incontinence during the 2nd week; however, the urinary incontinence symptoms were completely gone by the end of the 4th week. The remaining three patients developed urinary incontinence during the 8th week with a frequency of three times per week, 10 times per week, and five times per week, respectively; however, two out of these three had no incontinence during followup; the remaining one continued to present with urinary incontinence which was 5 times per week during followup.

### 3.2. Assessment of Bowel Function

Before treatment, all 14 patients had constipation and required supplementary methods for defecation, and thus their symptoms were considered as complete constipation. After treatment, four (4/14, 28.57%) resumed normal bowel movements; five (5/14, 35.71%) regained partially normal bowel movements and reduced the dependence on supplementary defecation methods; five (5/14, 35.71%) had no change. In the five patients who regained partially normal bowel movements, one resumed the desire to defecate, one resumed anal reflex, and three regained the soft and smooth stool which was made of nuts-like hard lumps before. The effects of EA were maintained during followup (Table 1).

### 3.3. Adverse Effects

No adverse effects were found. Five patients with residual sensory functions reported pain, sourness, numbness, and/or distension upon acupuncture procedures, which were well tolerated during treatment and disappeared after needle removal. Based on traditional Chinese medicine theories, these sensations are likely to be categorized into the De Qi sensations and are thus considered normal during acupuncture treatment [16].

## 4. Discussion

### 4.1. EA and Function Recovery in SCI Patients

Under physiological conditions, neurons are considered as permanent cells which remain in the G0 phase and lack proliferation. With spinal cord injury, sprouting and regeneration of the CNS tracts depend on the functions of glia cells due to the poor regenerative capacity of axons [17]. The development of a glial scar and the accumulation of extracellular matrix (ECM) proteoglycans generate an impermeable barrier that further hinders axons from regenerating across the site of injury [18, 19]. Thus, neurological dysfunctions caused by SCI are generally considered extremely difficult to recover [11]. Patients with SCI who have no perianal pinprick sensation at initial evaluation never regain volitional voiding and bowel function recovery following SCI is not favorable [20]. Therefore, therapies that enhance more natural patient control of voiding and defecation are appealing. In the present study, adult patients with a history of chronic neurogenic bowel and bladder symptoms were included. Improvements in bowel and bladder functions in these patients are more likely to be related to the therapeutic effects of EA rather than spontaneous recovery.

### 4.2. Bowel and Bladder Functions with EA

Improvements of bowel and bladder functions will reinforce patients' confidence in recovery, encourage patient's social interaction, and increase the quality of life and thus greatly decrease the incidence of SCI-associated complications and the development of other comorbidities [11]. Recently, sacral neuromodulation which employs implants of electric stimulator in the pelvic region and surgical reinnervation with nerve anastomosis were found effective in the treatment of bowel and bladder dysfunctions caused by TSCI [21–23]. However, these interventions require surgical procedures which may be only applicable to a limited number of patients and may be accompanied with unfavorable side effects [5]. EA at S2-3 provides a combinational stimulation of acupuncture and electric therapy; therefore, EA therapy in the present study can be considered as minimal invasive type of sacral neuromodulation which has minimal side effects.

Results of the present study indicate that EA at S2-3 and perianal region could improve patients' voiding abilities. The results were consistent with our previous findings in which 10 out of 15 patients with cauda equina injury resumed voiding abilities and were urinary catheter-free after a similar EA treatment protocol [10]. Honjo et al. [9] found that EA at S2 was found to be effective in improving bladder control decreasing incontinence frequencies in patients with detrusor hyperreflexia. The present study demonstrated a similar response of EA via decreasing patients' incontinence frequencies. In the present study, detrusor hyperreflexia in nine patients (five patients with previous detrusor hyperreflexia and four patients with transient detrusor hyperreflexia) was successfully treated by EA and had no urinary incontinence at the end of treatment. Furthermore, Wong et al. [8] found that EA could decrease incontinence frequencies or assistance for bowel care in patients with SCI; the results of the present study indicate that EA could increase patients' abilities to self-initiate bowel movements and decrease the need of supplementary defection methods. Thus, the results of the present study add further credence to the therapeutic effects of EA on neurogenic bowel.

Bladder functions can also be reflected by postvoid RUV. In the present study, postvoid RUV was measured using a cylinder via self-urethral catheterization after full urination. This technique theoretically provides reliable information regarding residue urine volume and thus the first impression of patients' voiding function [24]. In the present study, EA was found to significantly decrease postvoid RUV both after treatment and during followup. These results echo with our previous findings in patients with cauda equina injury patients [10]. The decrease in postvoid RUV reflects the improvement of voiding ability with EA in patients with neurogenic bladder. In addition, findings of heaven amount of postvoid RUV in patients with urinary incontinence or detrusor hyperreflexia in the present study indicate that involuntary contraction in urinary incontinence is ineffective which concurs with other urinary incontinence studies [25, 26].

### 4.3. Possible Therapeutic Mechanism of EA in Bowel and Bladder Dysfunctions

In conjuncture with evidence from the aforementioned research studies, we hypothesize that EA sacral nerve stimulation could modulate urinary functions in patients with SCI. Pelvic splanchnic nerves do not only provide parasympathetic innervations to the detrusor muscle responsible for involuntary bladder emptying, but also parasympathetic innervations to the sigmoid colon and rectum responsible for involuntary bowel movements. Somatic components of the pudendal nerve directly innervate the external urethral sphincter and the external anal sphincter via its branches of inferior anal nerves; normal functions of these sphincters serve as the foundation for normal bowel and bladder control. Both the pelvic splanchnic nerves and pudendal nerve arise from S2–S4; therefore, EA at S2-3 provides direct electric and mechanical stimulation to these nerves. Nonetheless, EA stimulation at S2-3 is nonselective which indicates two extremely opposite conditions. This could at least partially contribute to the interesting opposite phenomena (effects on neurogenic urinary retention and urinary incontinence) discovered with EA treatment at S2-3 [9, 10]. To better utilize this minimal invasive direct nerve stimulation, more studies are warranted to develop selective EA treatment on the parasympathetic nerves or the pudendal nerve.

### 4.4. Limitations

Nonetheless, the present study is a case series study with a relative small sample size which indicates that results of the present study may not well represent the general response of patients with chronic bowel and bladder dysfunction caused by SCI. For example, in the present study, standard deviations of some quantitative measurements (such as postvoid RUV and the change of weekly urinary incontinence frequency after treatment) were bigger than its corresponding mean which indicates relative wide dispersions of values in the small sample and is unfavorable for the statistical analyses. Assessments of acupuncture effects in the present study mainly depend on patients' reports which may be interfered with recall bias and patients' believes and emotional status upon visits. To objectively evaluate EA effects on neurogenic bowel and bladder dysfunction, we may need to incorporate urodynamic measurements in future studies. Furthermore, assessment of bowel functions is limited to patient-reported bowel movement frequencies and supplementary defecation methods, information like average time required for defecation, frequency of defecation, and frequency of fecal incontinence is lacking.

## 5. Conclusion 

In patients with chronic bowel and bladder dysfunction after SCI, EA may provide a valuable alternative tool in improving patients' self-controlled bowel and bladder functions.

## Figures and Tables

**Table 1:** Demographic information and changes of urination and defecation after electroacupuncture treatment.

No.	Gender (F/M)	Age (years)	Courses (months)	Injured level	AISA classification	Urination form			Defecation form		
						Baseline	after treatment	After followup	Baseline	after treatment	After followup
1	F	21	8	T11	A	Intermittent catheterization	Normal voiding	Normal voiding	Suppositories	Suppositories	Suppositories
2	F	36	6	L1	A	Intermittent catheterization	Normal voiding	Normal voiding	Suppositories + perianal stimulation	Suppositories + perianal stimulation	Suppositories + perianal stimulation
3	M	48	6	L1	B	Intermittent catheterization	Normal voiding	Normal voiding	Suppositories	Normal defecation	Normal defecation
4	M	25	12	L1	B	Intermittent catheterization	Partially normal voiding + pushing the bladder	Partially normal voiding + pushing the bladder	Suppositories + assisted by hands	Partially normal defecation + suppositories	Partially normal defecation + suppositories
5	F	22	11	C5	C	Intermittent catheterization	Partially normal voiding + intermittent catheterization	Partially normal voiding + intermittent catheterization	Suppositories + laxative	Partially normal defecation + suppositories	Partially normal defecation + suppositories
6	M	27	6	L1	C	Pushing the bladder	Partially normal voiding + pushing the bladder	Partially normal voiding + pushing the bladder	Suppositories	Normal defecation	Normal defecation
7	M	27	9	L1	B	Intermittent catheterization	Partially normal voiding + intermittent catheterization + pushing the bladder	Partially normal voiding + pushing the bladder	Suppositories	Suppositories	Suppositories
8	M	48	6	T10	B	Intermittent catheterization	Normal voiding	Normal voiding	Suppositories + laxative	Normal defecation	Normal defecation
9	F	43	14	T9	A	Pushing the bladder	Pushing the bladder	Pushing the bladder	Suppositories	Suppositories (dosage reduced ≥ 50%)	Suppositories (dosage reduced ≥ 50%)
10	M	38	14	C5	B	Intermittent catheterization	Partially normal voiding + intermittent catheterization	Partially normal voiding + intermittent catheterization	Suppositories	Suppositories (dosage reduced ≥ 50%)	Suppositories (dosage reduced ≥ 50%)
11	M	36	15	T3	C	Intermittent catheterization	Intermittent catheterization	Intermittent catheterization	Suppositories + laxative	Suppositories + laxative	Suppositories + laxative
12	F	26	7	T6	A	Pushing the bladder + intermittent catheterization	Pushing the bladder + intermittent catheterization	Pushing the bladder + intermittent catheterization	Suppositories	Suppositories	Suppositories
13	M	57	108	L1	B	Intermittent catheterization	Partially normal voiding + pushing the bladder	Partially normal voiding + pushing the bladder	Suppositories + laxative	Partially normal defecation + suppositories	Partially normal defecation + suppositories
14	M	26	6	T12	A	Intermittent catheterization	Pushing the bladder + intermittent catheterization	Intermittent catheterization	Suppositories	Normal defecation	Normal defecation

**Table 2:** Mean postvoid RUV.

*n*	Baseline (A)	After treatment (B)	Followup (C)	Difference *x* ± *s*			*t* value			*P* value		
				AB	AC	BC	AB	AC	BC	AB	AC	BC
14 (total)	262.43 ± 131.50	72.14 ± 83.59	63.57 ± 75.71	190.29 ± 101.87	198.86 ± 112.18	8.57 ± 26.56	6.989	6.633	1.207	0.000	0.000	>0.05
4 (group A)	210.00 ± 95.92	20.00 ± 18.26	17.50 ± 15.00	190.00 ± 108.62	192.50 ± 107.82	2.50 ± 5.00	3.498	3.571	1.000	0.040	0.038	>0.05
6 (group B)	222.23 ± 103.31	45.00 ± 32.71	43.33 ± 29.43	177.33 ± 112.01	179.00 ± 110.70	−1.67 ± 4.08	3.878	3.961	1.000	0.012	0.011	>0.05
4 (group C)	375.00 ± 155.46	165.00 ± 107.55	140.00 ± 108.32	202.50 ± 118.43	220.00 ± 163.10	10.00 ± 66.33	3.934	2.698	0.302	0.029	>0.05	>0.05
